# Neonatal Urine Metabolic Profiling and Development of Childhood Asthma

**DOI:** 10.3390/metabo9090185

**Published:** 2019-09-16

**Authors:** Bo L. Chawes, Giuseppe Giordano, Paola Pirillo, Daniela Rago, Morten A. Rasmussen, Jakob Stokholm, Klaus Bønnelykke, Hans Bisgaard, Eugenio Baraldi

**Affiliations:** 1 COPSAC, Copenhagen Prospective Studies on Asthma in Childhood, Herlev and Gentofte Hospital, University of Copenhagen, 2820 Copenhagen, Denmark; chawes@copsac.com (B.L.C.); daniela.rago@dbac.dk (D.R.); morten.arendt@dbac.dk (M.A.R.); stokholm@copsac.com (J.S.); kb@copsac.com (K.B.); bisgaard@copsac.com (H.B.); 2 Women’s and Children’s Health Department, University of Padova, 35128 Padova, Italy; giuseppe.giordano@unipd.it (G.G.); paola.pirillo@unipd.it (P.P.); 3 Fondazione Istituto di Ricerca Pediatrica, Città della Speranza, 35127 Padova, Italy; 4 Department of Pediatrics, Naestved Hospital, 4700 Naestved, Denmark

**Keywords:** asthma, childhood, metabolomics, neonate, urine, wheezing illnesses

## Abstract

Urine metabolomics case-control studies of childhood asthma have demonstrated a discriminative ability. Here, we investigated whether urine metabolic profiles from healthy neonates were associated with the development of asthma in childhood. Untargeted metabolomics by liquid chromatography-mass spectrometry was applied to urine samples collected at age 4 weeks in 171 and 161 healthy neonates born from mothers with asthma from the COPSAC2000 and COPSAC2010 cohorts, respectively, where persistent wheeze/asthma was prospectively diagnosed using a symptom-based algorithm. Univariate and multivariate analyses were applied to investigate differences in metabolic profiles between children who developed asthma and healthy children. Univariate analysis showed 63 and 87 metabolites (q-value < 0.15) in COPSAC2000 and COPSAC2010, respectively, which is promising for discriminating between asthmatic and healthy children. Of those, 14 metabolites were common among the two cohorts. Multivariate random forest and projection to latent structures discriminant analyses confirmed the discriminatory capacity of the metabolic profiles in both cohorts with estimated errors in prediction equal to 35% and AUCpred > 0.60. Database search enabled annotation of three discriminative features: a glucoronidated compound (steroid), 3-hydroxytetradecanedioic acid (fatty acid), and taurochenodeoxycholate-3-sulfate (bile acid). The urine metabolomics profiles from healthy neonates were associated with the development of childhood asthma, but further research is needed to understand underlying metabolic pathways.

## 1. Introduction

Childhood asthma is an inflammatory airway disorder, which has recently been increasing, with the prevalence more than doubling in Westernized societies worldwide over the past decades [1]. Half of young children experience asthma-like symptoms [2] and one-fifth of preschoolers develop recurrent asthma-like episodes [3], which is now the main reason for hospitalization, chronic medication usage, and repeated contact with health care providers with an associated large public health care expenditure [4]. Asthma is believed to originate earliest in the life course of genetically susceptible individuals as a result of complex gene–environment interactions causing immune deregulation, which leads to initiation of chronic inflammatory processes [5] that are detectable prior to symptom onset [6,7]. The presence of a low-grade disease activity in early life before symptoms emerge implicates the existence of biomarkers of perturbed biochemical pathways that may help understand the mechanisms behind why some children will develop wheezing disorders and asthma during childhood. So far, such biomarkers have not been identified.

Metabolomics is a high-dimensional method to assess the wide range of small-molecule metabolites in a biological sample, which may provide a hypothesis-free biomarker profile and simultaneously provides insight into perturbed metabolic pathways and underlying disease pathology [8]. Metabolomics profiling shows the current state of the metabolism ongoing in the biological system and is considered the omics platform most related to host phenotype as it represents the net result of gene–environment interactions [9]. Metabolomics has successfully been applied to asthma and studies have shown that both targeted and untargeted blood metabolomics profiles are capable of classifying case status [10] and are associated with phenotypic traits such as lung function [11]. In contrast to obtaining blood samples, urine is easy to collect in infants and young children and is usable for metabolomics profiling. Previous urine metabolomics studies of childhood asthma have demonstrated discriminative abilities in case-control settings [12,13] as well as an ability to predict whether a child with early life wheezing will outgrow the disease or go on to develop asthma [14]. However, no studies have investigated whether urine metabolomics profiles from healthy neonates are associated with development of asthma during childhood.

In this study, we conducted untargeted metabolomics profiling based on liquid chromatography-mass spectrometry of urine samples collected at age 4 weeks in children from the two independent Copenhagen Prospective Studies on Asthma in Childhood (COPSAC2000 and COPSAC2010) birth cohorts from Zealand, Denmark, to investigate the presence of asthma biomarkers and/or perturbed metabolic pathways before symptoms debut. The COPSAC2000 is a high-risk cohort of 411 children born to mothers with asthma [15], who at age 4 weeks received an oral dose of chloral hydrate prior to neonatal lung function testing [16], whereas the COPSAC2010 is a population-based cohort of 700 children [17]. In order to make the results comparable among the cohorts, we only selected urine samples for metabolomics analyses collected before intake of chloral hydrate from COPSAC2000 and only urine samples collected from children born to mothers with asthma from COPSAC2010.

## 2. Results

### 2.1. Baseline Characteristics

In the COPSAC2000, 171 children had an available urine sample collected before sedation with chloral hydrate. Of those 171 children, 20 (11.7%) developed persistent wheeze/asthma during their first 6 years of life. In the COPSAC2010 cohort, 161 children born to mothers with asthma had an available urine sample; 49 (30.4%) of those children developed persistent wheeze/asthma during their first 6 years of life. We considered the difference in sampling age between the two cohorts negligible (mean age of 35 days and range (22–76) days as 5th–95th percentile for the COPSAC2000 cohort, and mean age of 36 days and range (24–88) days for COPSAC2010).

### 2.2. Urine Metabolomics Analysis

The urine samples were analyzed using ultra-performance liquid chromatography coupled to quadrupole-time of flight mass spectrometry (UPLC-MS) operating in negative ionization mode. The UPLC-MS analysis of the urine samples led to the generation of two datasets, one for the COPSAC2000 and another for the COPSAC2010, both composed of 1555 Rt_m/z variables. The software Progenesis was used to process the UPLC-MS raw data (see Appendix A) and the obtained dataset was filtered, normalized, and scaled prior to analysis (see metabolomics datasets in Table S1 in the Supplementary Materials). A preliminary exploratory data analysis was performed by principal component analysis on each group, i.e., healthy children and children developing persistent wheeze/asthma in both cohorts, to identify outliers. No outliers were detected on the basis of the DModX test and Hotelling’s T2 test (significance level α = 0.05, see Figure S1 in the Supplementary Materials).

Thereafter, univariate analyses were conducted to assess the relationship between urine metabolic features and persistent wheeze/asthma status using *t*-tests with a false discovery rate (FDR) applied to the features showing a normal distribution (Shapiro–Wilk test *p*-value > 0.10) and the Mann–Whitney test with FDR for non-normally distributed data. The FDR was performed according to Storey [18] and features showing a q-value less than 0.15 were considered as characterizing children with versus without development of persistent wheeze/asthma. The receiver operating characteristic (ROC) curve analysis was used to characterize each feature by calculating the area under the curve (AUC) with a 95% confidence interval. A total of 63 relevant variables were selected in the COPSAC2000 dataset, whereas 87 were selected in the COPSAC2010 dataset. In Table 1 and Table 2, we report the statistics of the 27 features of the COPSAC2000 dataset and the 30 features of the COPSAC2010 dataset, which both showed q-values < 0.15 for both the *t*-test and the Mann–Whitney test; all variables showed significant AUC values (significance level α = 0.05).

Of the features selected by univariate analyses in COPSAC2000 and COPSAC2010, 14 variables were in common among the two datasets, i.e., discriminative of persistent wheeze/asthma development in both cohorts. In Table 3, we report the statistics of the 14 features selected in both the datasets. The complete results of the univariate data analysis are provided in Table S2 in the Supplementary Materials.

Projection to latent structures discriminant analysis based on variable influence on projection selection (VIP-based PLS-DA) was thereafter applied to investigate if the correlation structure in the metabolomics data was able to separate children who developed persistent wheeze/asthma from healthy children. Specifically, a stability selection procedure based on VIP-based PLS-DA was applied [19]; a total of 100 subsamples composed of 75% of the observations for the persistent wheeze/asthma group and an equal number of those for the healthy group were randomly selected from the samples. The relevant discriminant features were identified as those that were selected in more than 50% of all the models. The performance in prediction of each model was estimated by AUC of the prediction of the part of the samples that were not selected for training the model (AUCpred). The stability selection highlighted 11 discriminative features from the COPSAC2000 dataset and 35 discriminative features from the COPSAC2010 dataset. In Table 4, we provide a list of these variables. Variable 3.34_579.2516n was selected both by univariate and by multivariate data analysis in the COPSAC2010 cohort.

**Figure 1:**
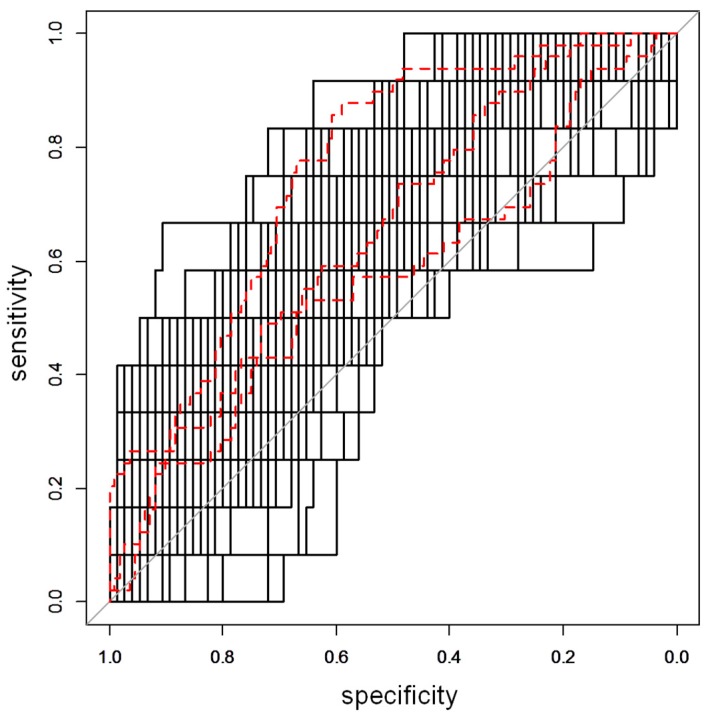
ROC curve for the COPSAC2010 dataset. In the plot, the 100 ROC curves calculated during the projection to latent structures discriminant analysis based on variable influence on projection selection (VIP-based PLS-DA) stability selection procedure for the COPSAC2010 dataset are reported, represented by the black lines. Red, dashed lines indicate the 10th, 50th, and 90th percentiles.

For the COPSAC2000 dataset, stability selection provided a model distribution characterized by a median AUCpred of 0.68 (10th percentile equal to 0.51) (see Figure S2 in the Supplementary Materials), whereas, for the COPSAC2010 dataset, the AUCpred was 0.63 (10th percentile equal to 0.54) (Figure 1). All the features selected as relevant were merged to the subset of features selected by the univariate data analyses. The confusion matrices obtained predicting the out-of-bag observations are reported in Tables S3 and S4 in the Supplementary Materials.

**Figure 2:**
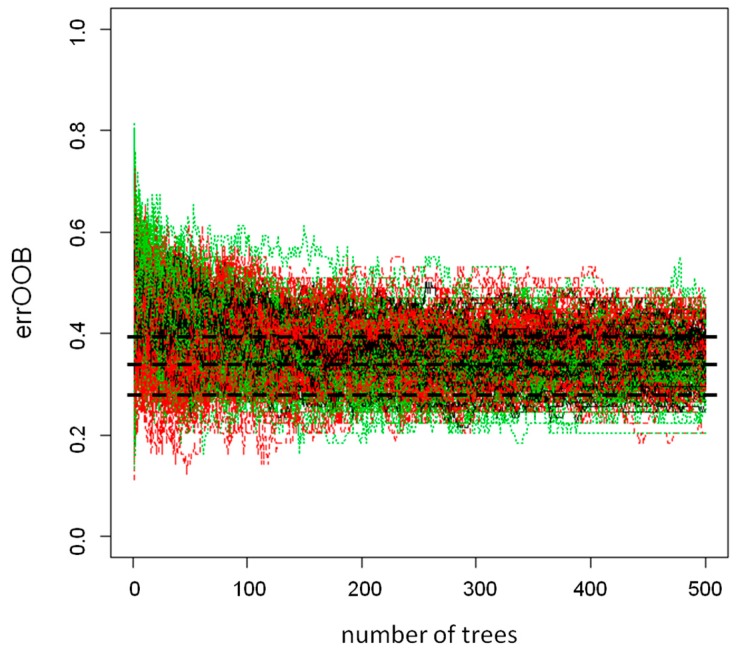
Random forest analysis of the COPSAC2010 dataset. Error-out-of bag (errOOB) versus the number of trees of the 500 random forests built on the COPSAC2010 dataset. Black, dashed lines indicate the 10th, 50th, and 90th percentiles of the errOOB. Red lines indicate the errOOB for the non-asthma group, the green lines that of the asthma group, and the black lines the total errOOB for each random forest. The total errOOB laid in the range of 0.28 to 0.39 after 300 trees. With more than 300 trees, the performance did not change.

The data were thereafter analyzed by random forest (RF) modeling [20] to assess if the metabolic fingerprint can be used to classify children who have developed persistent wheeze/asthma as well as healthy children. Since the asthma and the non-asthma group were not equally populated, we randomly extracted from the two groups 500 subsets composed of a number of observations equal to 70% of the less populated group. The extracted observations were used as training sets for RF, whereas the observations that were not selected were used to test the 500 RFs obtaining the error out-of-bag (errOOB). The RF built on the two datasets led to a median errOOB equal to 35% for both COPSAC2000 and COPSAC2010, according to the error in prediction estimated by the PLS-DA based models (Figure 2). The performance, in prediction of the classification models, allowed us to affirm that the metabolic fingerprints from urine samples of children aged 4 weeks contained information useful for characterizing the two groups under investigation, i.e., children developing persistent wheeze/asthma and healthy children.

### 2.3. Metabolite Annotation

To describe the relevant biology of the discriminative features for persistent wheeze/asthma status, metabolite annotation was attempted for the 14 features selected in COPSAC2000 and COPSAC2010. We searched our in-house database as well as the available online metabolite databases, the Human Metabolome Database (HMDB) and the METLIN Metabolite and Chemical Entity Database, and studied the fragmentation spectra, which led us to identify three relevant features. Two identified features in the COPSAC2010 cohort were higher in children developing persistent wheeze/asthma: the bile acid taurochenodeoxycholate-3-sulfate (3.34 Rt 579.2516n, annotation at level 1, as defined by the Chemical Analysis Working Group of the Metabolomics Standards Initiative [21]), and the fatty acid 3-hydroxytetradecanedioic acid (0.97 Rt 273.1696m/z, annotation at level 2). One feature in common with the two cohorts was higher in the healthy children and was annotated as a glucoronidated steroid compound (4.95 Rt 520.1083n, annotation at level 3) (see Appendix A).

## 3. Discussion

This pioneering urine metabolomics study of healthy neonates born to asthmatic mothers showed that differential urine metabolite levels measured at age 4 weeks in two birth cohorts were associated with development of asthma before school age. The metabolic profiles in the urine samples discriminated children developing asthma from healthy children independently in both cohorts and with 14 overlapping discriminating features. These findings suggest the presence of a perturbed metabolism in early life heralding the onset of respiratory symptoms.

Although no previous study has investigated urinary metabolomics profiles at age 4 weeks in relation to asthma development, other studies have shown that urine metabolomics profiles in the first years of life predict development of recurrent wheeze and asthma [22,23], which is in line with our findings. A recent longitudinal high-resolution ^1^H nuclear magnetic resonance (^1^H-NMR) spectroscopy study by Chiu et al. [22] of 60 children showed that the metabolic profiles in urine samples collected at ages 1–4 years was associated with asthma status by age 4. Dimethylamine, allantoin, guanidoacetic acid, and 1-methylnicotinamide from the nicotinamide pathway, which is associated with oxidative stress and smooth muscle cell contractility, were significantly associated with asthma development [22]. Another longitudinal ^1^H-NMR study by Turi et al. [23] of healthy infants and infants with respiratory syncytial virus (RSV) and rhinovirus infection showed that their urine metabolic profile at enrollment was associated with risk of recurrent wheezing through age three. They found that nicotinamide and nicotinate metabolites discriminated infants with RSV from healthy infants and that alanine and 2-hydroxyisobutyric acid, a gut microbiome metabolite, were associated with development of recurrent wheeze [23]. Interestingly, a case-control urine ^1^H-NMR study of 135 children aged 4 to 16 also found 1-methylnicotinamide and 2-hydroxyisobutyrate among other metabolites being able to separate asthmatics from healthy children [12].

In our UPLC-MS study, we were able to annotate three of the discriminatory features, which were the bile acid taurochenodeoxycholate-3-sulfate, the fatty acid 3-hydroxytetradecanedioic acid, and a glucoronidated steroid compound, but we were unable to annotate the majority of the features that were associated with development of persistent wheeze or asthma. The metabolite findings in the longitudinal studies by Chiu et al. and Turi et al. [22,23] were not among our annotated metabolites, but we could not determine whether our unknown discriminative features encompass these metabolites or other metabolites in the same pathways. However, lack of replication of metabolites between our study and their study could be caused by the young age of sampling in our study, the fact that we sampled urine from healthy neonates prior to any respiratory symptoms, and that profiling was done by UPLC-MS in negative ionization mode as opposed to ^1^H-NMR.

The bile acid metabolite taurochenodeoxycholate-3-sulfate is a sulfated bile salt formed in the liver by conjugation of chenodeoxycholate with taurine, which was a level 1 annotated identified metabolite that we found significantly higher in the urine of children developing asthma. Bile acids are known to facilitate the excretion, absorption, and transport of fats and sterols in the intestine and liver; they are essential for the absorption of the fat-soluble vitamins A, D, E, and K, and are involved in the regulation of enzymes in the cholesterol homeostasis. Sulfation of bile acids increases the aqueous solubility and results in increased renal excretion and decreased reabsorption from the intestinal lumen [24], which could be speculated to lead to decreased absorption of vitamin D and corresponding lower vitamin D blood levels, which have been associated with increased risk of childhood wheezing illnesses [25]. Furthermore, conjugated, non-sulfated bile acids such as tauroursodeoxycholic acid [26,27] and chenodeoxycholic acid [28] have, in murine models, been shown to attenuate allergic airway inflammation by inhibiting Th2 cytokines. Previous human urine metabolomics studies of asthma have not found bile acid metabolites associated with asthma status, but a plasma UPLC-MS case-control study of 237 children (mean age 8 years) from the American Project Viva birth cohort found that the bile constituent taurocholate significantly increased the risk of asthma [29]. A study by Comhair et al. [30] has also shown increased level of plasma taurocholate among asthmatics. In general, bile acids are emerging as regulators of the gut microbiome at the highest taxonomic levels. Recent evidence suggests crosstalk between gastrointestinal microbiota and the lung, as well as the existence of a gut-mediated lung immunity that could remotely contribute to the pathogenesis of chronic airway diseases such as childhood asthma [31,32,33].

The fatty acid 3-hydroxytetradecanedioic, which we found to be significantly higher in healthy neonates who developed persistent wheeze or asthma during their first six years of life, was annotated at level 2, i.e., a putatively annotated compound. Very high urinary levels of this unusual 3-hydroxydicarboxylic acid have been reported in infants with inborn errors of metabolism [34,35], but not in association with asthma. However, several targeted and untargeted urine and blood metabolomics studies, reviewed by Kelly et al. [10] and Turi et al. [36] have shown that lipid mediators and fatty acids are associated with asthma status, exemplified by McGeachie et al. [37] in a targeted blood lipidomics study of 20 children showing that monoHETE, arachidonic acid, and prostaglandin-E2 were associated with asthma control. Particularly, n-6 long-chained polyunsaturated fatty acids (LCPUFAs) related to arachidonic acid in membrane phospholipids are known to be implicated in airway inflammation and asthma pathogenesis via conversion to eicosanoids, leukotrienes, and prostaglandins [38], whereas n-3 LCPUFAs can displace arachidonic acid and dampen inflammation [39]. Thus, our findings in respect to 3-hydroxytetradecanedioic acid could be speculated to be a reflection of a more general perturbed pro-inflammatory fatty acid metabolism. However, that remains purely speculative, as the majority of the discriminative features could not be annotated in our study.

We also found a third significant discriminative feature at age 4 weeks that was higher in children not developing persistent wheeze or asthma. Database search and MS/MS fragmentation experiments on this feature only annotated the metabolite as a glucoronidated steroid compound, i.e., corresponding to level 3 putatively characterized compound classes and, therefore, this finding should be interpreted cautiously. Several metabolomics studies have shown that levels of endogenous steroids, such as cortisone in plasma [29,40] and urocortison and urocortisol in urine [13] are higher in healthy children compared to asthmatics, which is most likely caused by suppression of the hypothalamic–pituitary axis by maintenance treatment with inhaled corticosteroids. However, this cannot explain our findings as urine was sampled from healthy, asymptomatic four-week-old children free of any medical treatment. Glucuronidation is a process that assists in renal excretion of substances as it increases water solubility of the original substance. Therefore, it may be speculated that children developing persistent wheeze or asthma with lower level of the glucoronidated steroid compound in their urine at age 4 weeks had higher level of the original steroid substance in the blood, i.e., an increased stress response, which may have increased their risk of developing systemic low-grade inflammation [41] and asthma-like symptoms.

It is a significant strength of our study that we were able to show that, in the metabolomics profiles measured in urine samples from age 4 weeks, there were a metabolic fingerprints which were significantly associated with development of persistent wheeze and asthma in two independent birth cohorts with several overlapping features. However, from a clinical perspective, a prediction accuracy of around 0.65 for the multivariate models is not very convincing. Still, it is not too bad considering that the biological samples (urine) are not tissue specific for asthma and were collected years before diagnosis. The study is strengthened by the use of similar endpoints of persistent wheeze/asthma in the two birth cohorts strictly adhering to previously validated algorithm-based diagnostic procedures [42], which the limits diagnostic heterogeneity of childhood asthma seen in general practice and the recall bias inherent to using a parent history of doctor-diagnosed asthma. The generalizability of our findings is limited by the fact that all the samples selected for metabolomics profiling were collected from children born to mothers with a history of asthma.

Another strength of this study was the use of state-of-the-art UPLC-MS metabolic profiling. Still, we were unable to annotate the majority of the persistent wheeze/asthma discriminative features, which limits the biochemical insight into disease mechanisms obtained from the data. However, metabolite annotation difficulties with LC-MS in terms of identification of biochemical structures is a generic rather that study specific problem [43], and annotation of 3 of 14 common discriminating features is not less than would be expected. Metabolite libraries and computer-based interpretation of mass spectra data are rapidly improving, but exact metabolite annotation is a complicated procedure due to the complex patterns of fragmentations [44]. Thus, we were mainly able to discover a combination of unidentified features in the urine of children at 4 weeks of age, from which it was possible to discriminate between children developing persistent wheeze/asthma and children not developing such traits. Regardless of the fact that the biological natures of these features are mostly unknown, this is an important finding, as it pinpoints the existence of a perturbed metabolism earliest in the life course, which directs primary preventive initiatives such as dietary supplements to the pregnancy and early postnatal period.

## 4. Materials and Methods

### 4.1. Study Populations

The COPSAC2000 is a high-risk cohort including 411 children born to mothers with asthma. The symptom-free children were enrolled at age 4 weeks excluding children with gestational age < 36 weeks, any congenital abnormality, and any respiratory symptoms or treatment prior to enrollment. At the visit at age 4 weeks, the healthy neonates were sedated with an oral dose of chloral hydrate 90 mg/kg prior to lung function testing, with the raised volume rapid thoracoabdominal compression technique [16]. The children subsequently attended six monthly scheduled visits to the COPSAC research clinic until age 7, as well as immediately upon onset of any respiratory symptom [15]. Urine samples of 171 healthy neonates were collected before oral chloral hydrate for sedation and submitted to metabolomics analysis.

The COPSAC2010 is a population-based cohort of 738 women and their 700 children. The women were enrolled at 24 weeks of pregnancy, excluding women with any endocrine, heart, or kidney disorder or a vitamin D intake above 600 IU/d. The children were seen at scheduled visits at the COPSAC clinic at age 1 week; 1, 3, and 6 months, half-yearly till age 3; and yearly from ages 3–6, as well as during episodes with respiratory symptoms [17]. Two randomized controlled trials of fish oil and high-dose vitamin D during 3rd trimester pregnancy with asthma/persistent wheeze as primary endpoint were embedded in COPSAC2010 in a 2 × 2 factorial design [45,46]. A subgroup of 161 healthy neonates with high-risk of asthma, being born to mothers with asthma, was selected for metabolomics investigation.

The Local Ethics Committee (H-B-2008-093) and the Danish Data Protection Agency (2015-41-3696) approved the study. Both parents gave oral and written informed consent before enrolment.

### 4.2. Persistent Wheeze and Asthma Diagnosis

Parents were instructed to record their child’s troublesome lung symptoms, i.e., significant cough or wheeze or dyspnea, in a diary filled from birth as a dichotomized daily score (yes/no). Upon onset of symptoms, the parents were further requested to bring their child to the research unit for a clinical examination by the COPSAC pediatricians and verification of the diary recordings. The COPSAC pediatricians were solely responsible for diagnosis and treatment of asthma in the cohorts instead of their general practitioners.

Persistent wheeze (0–3 y)/asthma (>3 y) was diagnosed based on a previously validated quantitative symptom-based algorithm [42,47] requiring all of the following criteria: (1) five episodes of diary-verified troublesome lung symptoms within six months, each lasting at least three consecutive days; (2) symptoms typical of asthma, including exercise-induced symptoms, prolonged nocturnal cough, and persistent cough outside common cold; (3) need for intermittent rescue use of inhaled β2-agonist; and (4) response to a 3 month course of inhaled corticosteroids (ICS) and relapse upon ended treatment, which resulted in prescription of a 6 months course of ICS and additional 12 month courses at subsequent relapses.

The primary endpoint for the urine metabolomics analyses was development of persistent wheeze/asthma in the first 6 years of life.

### 4.3. Urine Collection and Sample Preparation

Urine samples were collected during scheduled visits to the COPSAC clinic at age 4 weeks into a sterile plastic bag adherent to the skin. The urine samples were immediately transferred to 3.6 mL Nunc tubes, and the aliquots were stored without addition of any preservatives at −80 °C until analysis [6].

The samples were shipped on dry ice to the Mass Spectrometry Laboratory of the Women’s and Children’s Health Department of Padova University Hospital. All urine samples were analyzed at the same time using UPLC coupled to quadrupole-time of flight mass spectrometry (Acquity coupled to Synapt G2; Waters). Firstly, urine samples were slowly thawed, in order to reduce thermal shock, which may alter the metabolic composition of the samples; their temperature were raised from −80 °C to −20 °C, then from −20 °C to +4 °C, and finally from +4 °C to ambient temperature (around +20 °C). Then, each sample was stirred and centrifuged at 3600× *g* for 10 min in order to make each sample more homogeneous and separate any solid particulate. Following centrifugation, 100 μL of the supernatant was transferred into a test tube and 200 μL of a solution of acetonitrile (CH_3_CN) and H_2_O in 90:10 ratio with 0.1% formic acid (FA) was added; thus, diluting the sample by a ratio of 1:3. After mixing the solution, 100 μL of the diluted sample was pipetted into a 384 well plate for the analysis in positive ionization mode, and another 100 μL was pipetted into a second 384 well plate for analysis in a negative ionization mode.

In order to assess the reproducibility and accuracy during the analysis, quality control samples (QC) and standards solution samples (Mix) were used. The QCs were prepared mixing together an aliquot of each sample and then making a 1:3 dilution (CH_3_CN:H_2_O, 0.1% FA). The standards solution consisted of a mix of nine compounds, where the exact masses and retention times were known (sulfaguanidine, scetaminophen, saffeine, hippurate, leucine enkephalin, sulfadimethoxine, verapamil, terfenadine, cholic acid). The QC and Mix samples were injected at regular intervals during the sequence, together with blank samples (CH_3_CN:H_2_O, 0.1% FA), to determine specific ions from the mobile phase and to see possible contaminants.

For all transfers of liquids, an Eppendorf micropipette was used to minimize contamination. The samples were injected randomly to prevent any spurious classification derived from the samples position in the sequence.

### 4.4. UPLC-MS Analysis

The well plates were put in the auto-sampler of the UPLC, which was kept at a temperature of 6 °C. A Waters Acquity BEH HILIC, with a 2.1 mm wide and a 100 mm long column packed with 1.7 μm beads, was used for normal stationary phase injection. The temperature of the column was kept at 50 °C and the mobile phase flow rate was set at 0.5 mL/min.

The gradient mobile phase consisted of H_2_O with 0.1% FA and 10 mM of ammonium acetate (CH_3_COONH_4_) for the eluent A and H_2_O with CH_3_CN in a 0.5:99.5 ratio with 0.1% FA and CH_3_COONH_4_ 1 mM for the eluent B. Each sample run lasted 12 min and consisted of an isocratic phase of 99% B for 1 min, a linear decrease to 80% B in 4.5 min, a linear decrease to 60% B in 0.5 min, a linear decrease to 5% B in 0.1 min, an isocratic phase of 5% B for 1.49 min, a linear increase to 99% B in 3.5 min. For each run, 5 μL of sample were injected.

Mass spectrometry and exact mass acquisition was performed with the quadrupole-time-of-flight mass spectrometer operating at negative-ion (ESI-) electro-spray ionization mode. We limited the analysis to metabolites falling in the mass range of 20 to 1200 amu. The capillary voltage was set at 1.5 KV. The desolvation gas flow was set at 600 L/h with the temperature kept at 350 °C. The cone gas flow was set at 20 L/h with the temperature kept at 110 °C.

To further reduce analytical variability, in accordance with an in-house protocol, sample distribution in the plate and the sequence of sample injection in the UPLC-MS were randomized, and five-sixths of the fluid resulting from the addition of eluents to the sample was excluded from the ionization process (splitting). Splitting samples prevents the risk of smudging the internal surfaces of the spectrometer itself, thus reducing its sensitivity. The analytical timeframe of all the analyses was within a month.

### 4.5. Data Processing and Pre-Treatment

The UPLC-MS data were processed by the software Progenesis V2.3 (Nonlinear Dynamics-Waters). The parameters used for data extraction were optimized through the preliminary analysis processing of the QC samples. As a result, the so called Rt_m/z variables (where Rt is the retention time and m/z is the mass to charge ratio of the chemical compound) were generated.

Variables with more than 95% of missing values in the blank samples or with a ratio between the 5th percentile measured in the QCs and the 95th percentile measured in the blank samples greater than 5 were included. Moreover, variables with a coefficient of variation in the QCs greater than 20% were excluded.

Missing data were imputed by generating a random number between zero and the minimum value measured for the variable. Probabilistic quotient normalization and square root transformation followed by mean centering and Pareto scaling were applied in order to take into account dilution effects and to reduce the scale effects of the variables (see Figure S3 in the Supplementary Materials).

### 4.6. Statistical Data Analysis

The relationships between the measured variables and the outcome of asthma were investigated using different methods in order to discover the most interesting variables able to characterize the urines of the subjects developing asthma with respect to the urines of the group that did not develop asthma. Specifically, *t*-test with FDR was applied to the variables showing a normal distribution in each group on the basis of the Shapiro–Wilk test (significance level of 0.10), whereas the Mann–Whitney test with FDR was applied to all datasets. The ROC curve analysis was used to characterize the strength in discrimination of each variable. The FDR was performed according to Storey [18] and the significance level for the q-value was set to 0.15.

To investigate the correlation structure related to the differences between the two groups, a stability selection procedure based on random sampling and VIP-based PLS-DA was implemented. The idea underlying stability selection is that real differences should be present consistently and, therefore, should be found even under perturbation of the data by sub-sampling. A subset of observations was randomly extracted and used to train a VIP-based PLS-DA model. In VIP-based PLS-DA, variables are selected excluding the variables showing a VIP score under a specified threshold that is calculated maximizing the cross-validated *R*^2^ obtained by 7 fold cross-validation. The procedure is repeated a large number of times, and variables are considered relevant on the basis of the number of VIP-based PLS-DA models that use those variables. Specifically, in both cohorts, a total of 100 subsamples composed of 75% of the observations for the persistent wheeze/asthma group and an equal number of those for the healthy group were randomly extracted from the collected samples, and then VIP-based PLS-DA was applied to each subsample, obtaining a set of 100 discriminant models. The relevant discriminant variables were identified as those that were selected in more than 50% of all the models. The predictive performance of each model was estimated by means of the analysis of the outcomes of the predictions of which samples would be excluded during sampling and expressed in terms of AUCpred. The class membership was determined applying linear discriminant analysis to the scores of the PLS-DA model.

In the case of RF, since the asthma and the non-asthma group were not equally populated, 500 subsamples were randomly extracted from the collected samples and RF was applied to each subsample. Specifically, 28 observations for the COPSAC2000 dataset (14 observations for the asthma group and 14 for the non-asthma group) and 68 observations for the COPSAC2010 dataset (34 observations for the asthma group and 34 for the non-asthma group) were randomly extracted to train 500 RFs. The out-of-bag error, i.e., the error calculated predicting the observations that were not selected as training sets, of each RF (500 trees, 40 candidate variables at each split) was considered an estimate of the error in prediction.

All analyses were implemented by the R 3.5.2 platform (R Foundation for Statistical Computing) using user-defined R-functions and the package “randomForest” for RF.

## 5. Conclusions

This exploratory study showed that in two independent birth cohorts, there were differential metabolite levels detected in urine samples between newborns that will develop asthma and those that will not; however, further research is needed to assess whether metabolic profiling of urine samples can accurately predict future asthma in a clinical setting.

## Figures and Tables

**Table 1:** Univariate data analysis results for COPSAC2000 **^1^**.

Metabolite ID		Higher in asthma/wheeze ^3^	*t*-Test *p*-Value	Mann–Whitney Test *p*-Value	AUC ^4^
Rt ^2^	Mass				
2.38	425.0667 m/z	N > Y	8.1 × 10^-11^	3.9 × 10^-7^	0.849
1.77	215.0312 m/z	N > Y	1.7 × 10^-9^	1.2 × 10^-7^	0.864
1.77	102.0554 m/z	N > Y	7.9 × 10^-7^	5.3 × 10^-4^	0.739
4.98	394.1348 m/z	Y > N	1.2 × 10^-5^	1.0 × 10^-3^	0.726
2.77	278.1021 m/z	Y > N	1.3 × 10^-5^	9.2 × 10^-6^	0.805
0.85	187.0604 m/z	Y > N	3.3 × 10^-5^	8.1 × 10-^4^	0.731
3.87	506.2357 n ^5^	Y > N	3.8 × 10^-5^	2.6 × 10^-3^	0.707
1.92	232.0570 m/z	Y > N	2.0 × 10^-4^	3.6 × 10^-3^	0.700
0.77	266.6361 n	N > Y	3.1 × 10^-4^	1.9 × 10^-4^	0.757
0.77	615.2553 m/z	N > Y	6.0 × 10^-4^	6.0 × 10^-4^	0.736
1.93	91.0519 n	Y > N	6.1 × 10^-4^	4.3 × 10^-3^	0.696
2.63	240.0830 n	N > Y	6.6 × 10^-4^	1.1 × 10^-3^	0.725
1.81	322.2205 n	Y > N	8.0 × 10^-4^	3.4 × 10^-3^	0.701
4.95	520.1083 n	N > Y	8.6 × 10^-4^	1.9 × 10^-3^	0.714
1.80	394.1729 n	Y > N	9.4 × 10^-4^	1.0 × 10^-3^	0.726
0.77	614.2596 m/z	N > Y	1.4 × 10^-3^	2.5 × 10^-3^	0.708
1.92	308.1753 n	Y > N	1.4 × 10^-3^	8.2 × 10^-3^	0.682
4.95	472.0821 n	N > Y	2.4 × 10^-3^	1.4 × 10^-3^	0.720
4.95	425.0693 m/z	N > Y	2.9 × 10^-3^	5.5 × 10^-3^	0.691
1.80	417.1577 m/z	Y > N	4.1 × 10^-3^	6.7 × 10^-3^	0.687
3.10	200.0651 m/z	Y > N	4.4 × 10^-3^	5.0 × 10^-3^	0.693
1.90	277.1282 m/z	Y > N	4.7 × 10^-3^	3.1 × 10^-3^	0.703
1.86	376.1704 n	Y > N	5.8 × 10^-3^	1.1 × 10^-3^	0.725
1.94	249.1164 n	Y > N	5.9 × 10^-3^	7.4 × 10^-3^	0.684
1.84	384.1464 m/z	Y > N	1.5 × 10^-2^	6.5 × 10^-3^	0.687
1.93	238.1074 m/z	Y > N	2.2 × 10^-2^	7.4 × 10^-3^	0.684
2.34	320.0695 n	N > Y	2.6 × 10^-2^	5.6 × 10^-3^	0.691

^1^ COPSAC2000 is the Copenhagen Prospective Studies on Asthma in Childhood 2000 birth cohort. ^2^ Rt is retention time. ^3^ Y > N indicates metabolites that show higher levels in the asthma group than in the non-asthma group, whereas N > Y indicates metabolites showing higher levels in the non-asthma group than in the asthma group. ^4^ AUC is the area under the ROC curve. ^5^ The metabolite masses with “n” indicate the neutral mass derived from the sum of relative intensity of some possible adducts, e.g., M+H, M+NH_4_, M+Na, and M+K, in the peak picking.

**Table 2:** Univariate data analysis results for COPSAC2010 **^1^**.

Metabolite ID		Higher in asthma/wheeze ^3^	*t*-Test *p*-Value	Mann–Whitney Test *p*-Value	AUC ^4^
Rt ^2^	Mass				
0.83	197.0309 m/z	N > Y	5.7 × 10^-9^	7.9 × 10^-7^	0.745
3.34	579.2516 n ^5^	Y > N	5.2 × 10^-6^	2.8 × 10^-4^	0.680
3.18	287.6108 m/z	Y > N	8.9 × 10^-6^	3.0 × 10^-5^	0.707
0.75	526.1535 n	Y > N	3.0 × 10^-5^	8.2 × 10^-4^	0.666
0.77	266.6361 n	N > Y	4.8 × 10^-5^	1.0 × 10^-3^	0.663
0.97	113.0466 n	Y > N	6.6 × 10^-5^	7.7 × 10^-5^	0.696
0.97	273.1696 m/z	Y > N	2.9 × 10^-4^	1.3 × 10^-3^	0.659
0.75	524.1340 n	Y > N	3.5 × 10^-4^	7.7 × 10^-4^	0.666
2.51	296.0547 m/z	Y > N	3.7 × 10^-4^	7.2 × 10^-4^	0.668
2.42	507.2270 m/z	N > Y	4.7 × 10^-4^	5.5 × 10^-3^	0.637
1.86	376.1704 n	Y > N	5.2 × 10^-4^	7.6 × 10^-3^	0.632
1.82	310.1155 m/z	Y > N	6.8 × 10^-4^	1.1 × 10^-3^	0.661
4.88	1022.5875 m/z	N > Y	6.9 × 10^-4^	2.8 × 10_-3_	0.648
4.22	568.1558 m/z	N > Y	7.1 × 10^-4^	3.0 × 10^-3^	0.647
6.82	298.0351 m/z	N > Y	7.7 × 10^-4^	1.7 × 10^-3^	0.656
1.19	365.1172 m/z	N > Y	1.1 × 10^-3^	4.5 × 10^-3^	0.641
4.88	938.6252 m/z	N > Y	1.2 × 10^-3^	3.7 × 10^-3^	0.644
2.55	185.0551 m/z	Y > N	1.2 × 10^-3^	5.9 × 10^-3^	0.636
1.78	159.0757 n	N > Y	1.2 × 10^-3^	2.2 × 10^-3^	0.652
0.76	509.1502 m/z	Y > N	1.4 × 10^-3^	7.5 × 10^-3^	0.633
6.84	268.1436 n	N > Y	1.6 × 10^-3^	3.0 × 10^-3^	0.647
0.75	254.0712 m/z	Y > N	2.4 × 10^-3^	9.5 × 10^-3^	0.628
4.10	399.0603 m/z	Y > N	4.2 × 10^-3^	8.7 × 10^-3^	0.630
0.99	626.2139 m/z	Y > N	4.8 × 10^-3^	7.6 × 10^-3^	0.632
0.76	561.2416 n	Y > N	4.9 × 10^-3^	2.1 × 10^-3^	0.652
0.76	269.0584 m/z	Y > N	5.4 × 10^-3^	8.7 × 10^-3^	0.630
2.25	90.0214 n	N > Y	6.3 × 10^-3^	4.3 × 10^-3^	0.641
0.75	253.5653 m/z	Y > N	1.1 × 10^-2^	7.2 × 10^-3^	0.633
4.87	633.7972 n	N > Y	1.3 × 10^-2^	8.5 × 10^-3^	0.630
5.07	307.1007 m/z	Y > N	1.4 × 10^-2^	5.7 × 10^-3^	0.637

^1^ COPSAC2010 is the Copenhagen Prospective Studies on Asthma in Childhood 2010 birth cohort. ^2^ Rt is retention time. ^3^ Y > N indicates metabolites that show higher levels in the asthma group than in the non-asthma group, whereas N > Y indicates metabolites showing higher levels in the non-asthma group than in the asthma group. ^4^ AUC is the area under the ROC curve. ^5^ The metabolite masses with “n” indicate the neutral mass derived from the sum of relative intensity of some possible adducts, e.g., M+H, M+NH_4_, M+Na, and M+K, in the peak picking.

**Table 3:** Univariate data analysis results for variables selected in both COPSAC **^1^** cohorts.

Metabolite ID		Higher in asthma/wheeze ^3^	COPSAC2000 ^4^	COPSAC2010 ^4^
Rt ^2^	Mass			
0.83	197.0309 m/z	N > Y	2.5 × 10^-4^	7.9 × 10^-7^
4.95	520.1083 n ^5^	N > Y	1.9 × 10^-3^	1.8 × 10^-4^
2.63	240.0830 n	N > Y	1.1 × 10^-3^	5.9 × 10^-4^
2.62	323.1058 m/z	N > Y	1.3 × 10^-6^	6.2 × 10^-4^
0.77	266.6361 n	N > Y	1.9 × 10^-4^	1.0 × 10^-3^
4.95	425.0693 m/z	N > Y	5.5 × 10^-3^	1.7 × 10^-3^
4.95	472.0821 n	N > Y	1.4 × 10^-3^	3.3 × 10^-3^
2.34	320.0695 n	N > Y	5.6 × 10^-3^	4.0 × 10^-3^
2.84	671.1053 m/z	N > Y	1.3 × 10^-4^	8.1 × 10^-3^
2.84	503.0769 m/z	N > Y	1.3 × 10^-4^	8.9 × 10^-3^
2.02	314.0735 m/z	Y > N	9.4 × 10^-4^	6.9 × 10^-4^
5.07	480.2963 m/z	Y > N	7.8 × 10^-4^	4.2 × 10^-3^
0.95	611.2039 m/z	Y > N	8.2 × 10^-4^	4.1 × 10^-3^
1.86	376.1704 n	Y > N	1.1 × 10^-3^	7.6 × 10^-3^

^1^ COPSAC is the Copenhagen Prospective Studies on Asthma in Childhood. ^2^ Rt is retention time. ^3^ Y > N indicates metabolites that show higher levels in the asthma group than in the non-asthma group, whereas N > Y indicates metabolites showing higher levels in the non-asthma group than in the asthma group. ^4^ Mann–Whitney test *p*-values are given in the COPSAC2000 and COPSAC2010 columns. ^5^ The metabolite masses with “n” indicate the neutral mass derived from the sum of relative intensity of some possible adducts, e.g., M+H, M+NH_4_, M+Na, and M+K, in the peak picking.

**Table 4:** Variable selected by stability selection.

Dataset	Metabolite ID		*N* ^2^
	Rt ^1^	Mass	
COPSAC2010	6.52	130.9831 m/z	77
COPSAC2010	6.46	164.9354 m/z	91
COPSAC2010	5.77	171.0055 m/z	52
COPSAC2010	0.83	197.0309 m/z	81
COPSAC2010	6.82	208.9162 m/z	60
COPSAC2010	6.75	239.0211 n ^3^	52
COPSAC2010	6.51	272.9581 m/z	91
COPSAC2010	6.47	278.9172 m/z	85
COPSAC2010	6.68	288.9358 m/z	81
COPSAC2010	3.80	303.6059 m/z	66
COPSAC2010	6.69	304.9131 m/z	75
COPSAC2010	6.69	273.9368 n	88
COPSAC2010	6.40	279.8984 n	74
COPSAC2010	6.52	327.0678 m/z	77
COPSAC2010	4.95	329.1580 n	54
COPSAC2010	6.65	357.0611 m/z	86
COPSAC2010	6.83	386.1231 n	56
COPSAC2010	6.65	372.0835 n	84
COPSAC2010	6.75	368.9785 n	70
COPSAC2010	6.69	434.8710 m/z	89
COPSAC2010	3.31	452.3010 m/z	72
COPSAC2010	6.62	472.2043 m/z	56
COPSAC2010	5.07	480.2963 m/z	84
COPSAC2010	2.51	534.1231 m/z	55
COPSAC2010	3.94	544.2579 m/z	55
COPSAC2010	4.95	520.1083 n	52
COPSAC2010	3.34	579.2516 n	57
COPSAC2010	0.94	595.2081 m/z	70
COPSAC2010	0.95	611.2039 m/z	70
COPSAC2010	6.65	305.0493 n	79
COPSAC2010	6.59	888.2750 m/z	84
COPSAC2010	6.40	891.1497 m/z	52
COPSAC2010	6.58	915.2940 m/z	70
COPSAC2010	2.84	1007.1621 m/z	56
COPSAC2010	2.84	1091.1767 m/z	55
COPSAC2000	1.77	215.0312 m/z	80
COPSAC2000	5.07	480.2963 m/z	50
COPSAC2000	2.84	503.0769 m/z	53
COPSAC2000	0.94	595.2081 m/z	63
COPSAC2000	0.51	655.0471 m/z	51
COPSAC2000	2.84	671.1053 m/z	59
COPSAC2000	2.83	839.1337 m/z	70
COPSAC2000	2.84	923.1478 m/z	69
COPSAC2000	2.84	1007.1621 m/z	83
COPSAC2000	2.84	1091.1767 m/z	74
COPSAC2000	2.83	1175.1912 m/z	79

^1^ Rt is retention time. ^2^ Number of sub-models where the variable was selected by projection to latent structures discriminant analysis based on variable influence on projection selection (VIP-based PLS-DA). ^3^ The metabolite masses with “n” indicate the neutral mass that derives from the sum of relative intensity of some possible adducts, eg, M+H, M+NH_4_, M+Na, and M+K, in the peak picking.

## Supplementary Materials

The following are available online at https://www.mdpi.com/2218-1989/9/9/185/s1, Figure S1: T2 versus DModX plots of the PCA models used for detecting outliers, Figure S2: ROC curve for the COPSAC2000 dataset, Figure S3: PCA models of the data before and after Probabilistic Quotient Normalization, Table S1: Metabolomics datasets, Table S2: Full univariate analyses, Table S3: COPSAC2000 confusion matrix, Table S4: COPSAC2010 confusion matrix.

## Appendix A

**Levels used for metabolite annotation according to Sumner et al. Metabolomics 2007, *3*, 211–221.**

**Level 1** “Identified metabolites” Two orthogonal analytical techniques applied to the analysis of both the metabolite of interest and to a chemical reference standard of suspected structural equivalence, with all analyses performed under identical analytical conditions within the same laboratory.

**Level 2** “Putatively annotated compounds” As for levels 3 and 4, including spectral (NMR and/or MS) similarity with public or commercial libraries.

**Level 3** “Putatively characterized compound classes” As for level 4, plus spectral and/or physicochemical properties consistent with a particular class of organic compounds.

**Level 4** “Unknown” A discernible spectral signal (NMR, MS or other) that can be reproducibly detected and quantified.

**Parameters used for data extraction by Progenesis 2.3**

Import the Data

○ Filter was set 0.25 on POS and NEG acquisition
○ Adducts
  ○ POS (M+H, M+Na, M+K, M+H-H2O, M+NH_4_, M+CH_3_OH+H)
  ○ NEG (M-H, M+Na-_2_H, M-H_2_O-H, M-NH_4_, M+CH_3_CO_2_)

Automatic alignment

Sensitivity was set to 3

Compounds Identify

Search to identity compounds using Progenesis on Home Database

○ Filter 10 ppm for mass
○ Filter 0.5 min for Rt
○ Filter 20 ppm for mass fragment

Search to identity compounds using METLIN, HMDB, as the databases

○ Filter 10 ppm for mass
○ Filter 20 ppm for mass fragment

All the compounds found were manually controlled on raw data.
