# Supplementary material for: Neonatal Urine Metabolic Profiling and Development of Childhood Asthma
Source: Metabolites. 2019 Sep 16;9(9):185. doi: 10.3390/metabo9090185 (PMC6780518; doi:10.3390/metabo9090185)
Supplement: Supplementary file 1 [file metabolites-09-00185-s001.zip › metabolites-548653-Supplementary Material.pdf]

metabolites

SUPPLEMENTARY MATERIAL

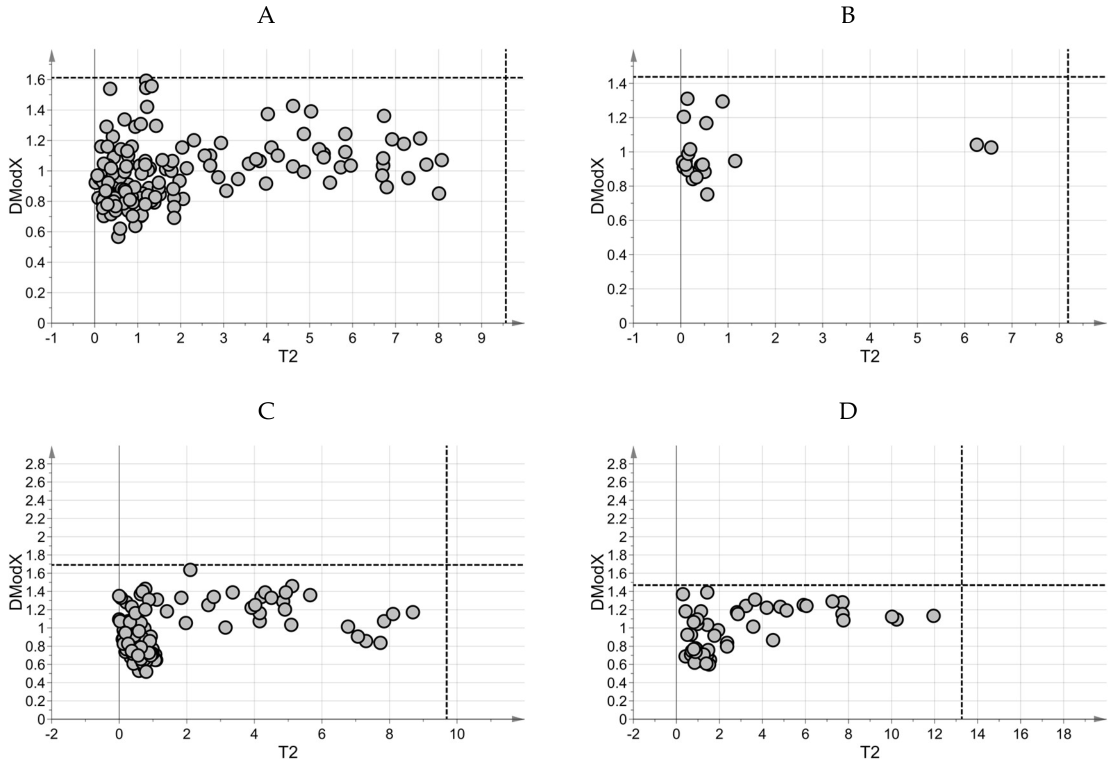

Figure S1. T2 vs DModX plots of the PCA models used for detecting outliers: no asthma group data set COPSAC2000 (R^2=0.56, panel A); asthma group COPSAC2000 (R^2=0.61, panel B); no asthma group COPSAC2010 (R^2=0.51, panel C); asthma group (R^2=0.58, panel D). Dashed lines indicate the limits at the significance level α=0.05.

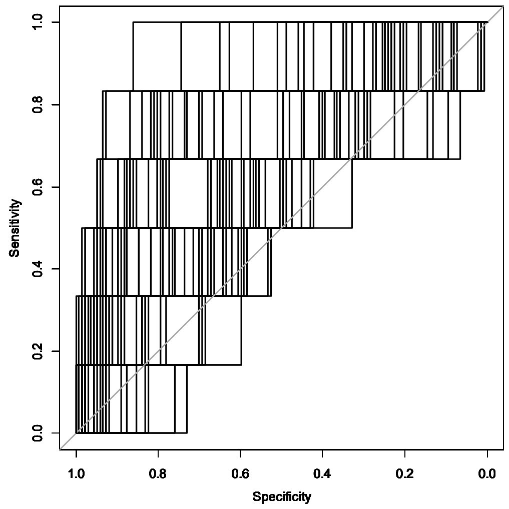

Figure S2. ROC curve for the COPSAC2000 data set. In the plot the 100 ROC curves calculated during the VIP-based PLS-DA stability selection procedure for the COPSAC2000 data set are reported.

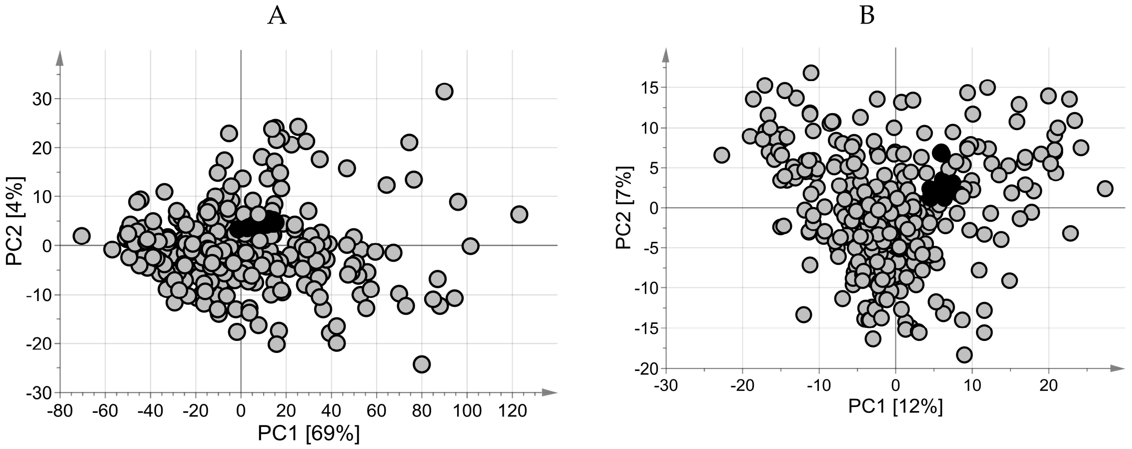

Figure S3. PCA models of the data before and after Probabilistic Quotient Normalization. In the score scatter plots QCs are reported as black circles whereas samples as grey circles. In both plots, QCs lie close together near to the center, whereas the samples occupy a larger region of the plot. After data normalization, samples occupy a reduced region because the effects of sample dilution were removed.

Table S3. COPSAC2000 data set: confusion matrix obtained by stability selection predicting the out-of-bag observations.

	pred no asthma	pred asthma
no asthma	134	17
asthma	16	4

Table S4. COPSAC2010 data set: confusion matrix obtained by stability selection predicting the out-of-bag observations.

	pred no asthma	pred asthma
no asthma	88	24
asthma	26	23
